# Nanocellulose Removes the Need for Chemical Crosslinking in Tannin-Based Rigid Foams and Enhances Their Strength and Fire Retardancy

**DOI:** 10.1021/acssuschemeng.2c02678

**Published:** 2022-07-25

**Authors:** André Luiz Missio, Caio G. Otoni, Bin Zhao, Marco Beaumont, Alexey Khakalo, Tero Kämäräinen, Silvia H. F. Silva, Bruno D. Mattos, Orlando J. Rojas

**Affiliations:** † Graduate Program in Materials Science and Engineering (PPGCEM), Federal University of Pelotas (UFPel), Gomes Carneiro 1, Pelotas, RS 96010-610, Brazil; ‡ Department of Materials Engineering (DEMa), Federal University of São Carlos (UFSCar), Rod. Washington Luís km 235, São Carlos, SP 13565-905, Brazil; § Department of Bioproducts and Biosystems, School of Chemical Engineering, Aalto University, Vuorimiehentie 1, Espoo FI-00076, Finland; ∥ Department of Chemistry, Institute of Chemistry of Renewable Resources, University of Natural Resources and Life Sciences, Konrad-Lorenz-Str. 24, 3430 Tulln, Austria; ⊥ VTT Technical Research Centre of Finland, P.O. Box 1000, Espoo FI-02044, Finland; # Bioproducts Institute, Department of Chemical and Biological Engineering, Department of Chemistry and Department of Wood Science, University of British Columbia, Vancouver, British Columbia V6T 1Z4, Canada

**Keywords:** condensed tannins, cellulose nanofibrils, nonstructural building materials, solid foams, thermal insulation, nonflammable foams

## Abstract

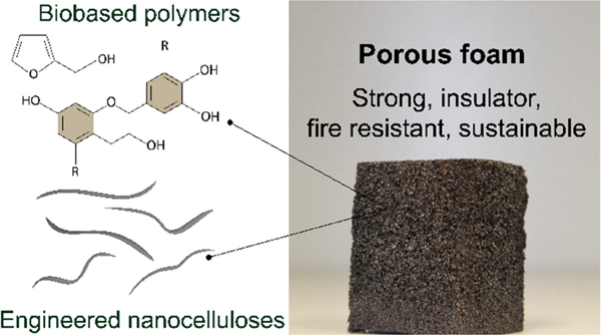

Thermal insulation and fire protection are two of the most critical features affecting energy efficiency and safety in built environments. Together with the associated environmental footprint, there is a strong need to consider new insulation materials. Tannin rigid foams have been proposed as viable and sustainable alternatives to expanded polyurethanes, traditionally used in building enveloping. Tannin foams structure result from polymerization with furfuryl alcohol via self-expanding. We further introduce cellulose nanofibrils (CNFs) as a reinforcing agent that eliminates the need for chemical crosslinking during foam formation. CNF forms highly entangled and interconnected nanonetworks, at solid fractions as low as 0.1 wt %, enabling the formation of foams that are *ca*. 30% stronger and *ca*. 25% lighter compared to those produced with formaldehyde, currently known as one of the best performers in chemically coupling tannin and furfuryl alcohol. Compared to the those chemically crosslinked, our CNF-reinforced tannin foams display higher thermal degradation temperature (peak shifted upward, by 30–50 °C) and fire resistance (40% decrease in mass loss). Furthermore, we demonstrate partially hydrophobized CNF to tailor the foam microstructure and derived physical–mechanical properties. In sum, green and sustainable foams, stronger, lighter, and more resistant to fire are demonstrated compared to those produced by formaldehyde crosslinking.

## Abstract 2

Rigid foams are made less toxic, more sustainable, and safer than their aldehyde-crosslinked analogues by using wood-derived tannin and nanocellulose.

## Introduction

Efficient thermal insulation is paramount to increase the energy performance in buildings^1,2^ as well as to reduce carbon emissions arising from artificial heating or cooling.^3^ For instance, the energy needed for achieving thermal comfort accounts to *ca.* 50% of the total residence energy consumption.^4^ Such high values remarkably impact the environmental footprint of buildings because the energy utilized in heating or cooling typically comes from nonrenewable sources.^5^ Moreover, heating appliances are known to be among the main culprits in residential fires. The latter account to over 300,000 cases per year, leading to civilian deaths and injuries (>10,000), as well as property damage (billions of US dollars).^6^ Therefore, both insulating and fire-retardant materials are key elements in building external and internal envelopes.

Polyurethane foams (PF) have been the material of choice for insulation purposes, typically applied via spray technologies.^7^ PF are produced by the reaction of isocyanates with polyols using amine-based catalysts. They are usually combined with flame retardants (e.g., halogenated compounds) given the PF flammability and the highly toxic and combustible gases it generates.^8−10^ Hence, despite PF’s excellent thermal insulation,^11,12^ there are major concerns related to safety and environmental impact.^7^ Therefore, many efforts have been directed to produce efficient thermal insulating and fire-resistant foams from eco-friendly and nonhazardous components.^13,14^ In this area, plant-based phenolic molecules, such as lignins^14^ and tannins,^15^ have been shown to potentially replace isocyanate PF. For instance, tannin-based rigid foams, built from the copolymerization of condensed tannins and furfuryl alcohol under acidic conditions,^16^ have been reported along with major advances in the optimization of foam synthesis^17,18^ and properties.^19,20^ However, these materials are still heavily dependent on chemical crosslinkers,^18,21^ such as formaldehyde and glutaraldehyde. They are used to strengthen the microstructure to meet the criteria required for technical applications. To further develop this area, new material-centered strategies are needed to completely remove hazardous chemical crosslinkers, while simultaneously keeping or enhancing the mechanical and insulating performance of the foam.

**Figure 1:**
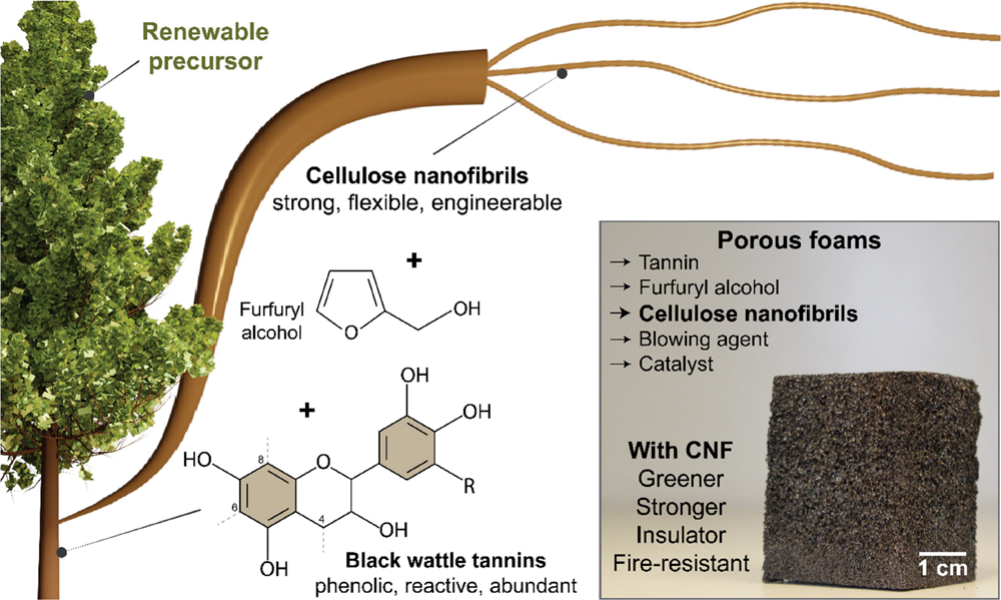
Left: Overall scheme of the preparation of tannin-furfuryl alcohol rigid foams reinforced with CNF. CNF is added as a wall reinforcer to replace chemical crosslinkers, here benchmarked by formaldehyde. The resulting CNF-containing tannin-furfuryl alcohol foams display thermal insulation capacity, fire self-extinguishing character, and are stronger and more sustainable than those containing chemical crosslinkers. Right: Porous foams are produced from plant-based building blocks. The chemical structure displayed represents a major group of compounds identified in black wattle tannins, including proanthocyanidin, which can polymerize from carbons 4, 6, and 8 to form a tridimensional macromolecule.^24−26^

In this work, rigid foams were prepared by unpurified black wattle tannin and furfuryl alcohol in the presence of cellulose nanofibers (CNF) at low loadings, removing the need for chemical crosslinking (Figure 1). CNF assemble into highly entangled nanonetworks, both in single-component and composite systems, enabling efficient stress transfer during mechanical solicitation.^22^ The dispersing and structuring capabilities of CNF also help tailor the morphology of the foam, which we discuss in terms of CNF surface activity after regioselective esterification.^23^ We show that the addition of CNF (*ca.* 0.1% based on total dry mass) leads to robust tannin rigid foams (Figure 1), stronger than the best performers obtained by chemical crosslinking.^15^ We evaluate the thermal insulating and fire-retardant properties of the tannin-based, CNF-reinforced foams, which are simultaneously stronger, lighter, and more sustainable compared to the nonbiogenic counterparts.

## Experimental Section

### Materials

The condensed tannins were extracted from the bark of black wattle (*Acacia mearnsii*) and supplied by TANAC (Brazil). Furfuryl alcohol (98%), diethyl ether (99.5%), *p*-toluene sulfonic acid (65% w/v), and formaldehyde solution (37% w/v) were purchased from Sigma-Aldrich. The properties of the condensed tannins are listed in Table S1. High-purity cellulose fibers were supplied by Lenzing AG (Lenzing, Austria), as never-dried bleached beech sulfite dissolving pulp (50% w/v). The cellulose fiber slurry was modified following our regioselective method for esterification of C6-OH with acetyl and isobutyryl groups, leading to acetylated and isobutyrylated CNF (CNF-AA and CNF-iBA, respectively).^23^ The fibers were diluted to 1% (w/v), blended in a high-shear homogenizer, and then fibrillated using a high-pressure fluidizer (Microfluidics M110P) using six passes (200 and 100 μm chambers at 2000 bar). The obtained nanofibers were cast into a film for chemical analyses by Fourier-transform infrared (FTIR) spectroscopy, recorded in the wavenumber range from 4000 to 600 cm^–1^ using a PerkinElmer spectrometer in attenuated total reflectance (ATR) mode with a resolution of 2 cm^–1^ using 20 scans for data acquisition. The films were also used to verify the success of the surface modification by assessing the water contact angle (WCA) with a sessile drop of deionized water on a goniometer (Biolin Scientific, Theta Flex) (Figure S1).

### Preparation of the CNF-Reinforced Tannin-Based Rigid Foams

For the preparation of the tannin rigid foam (in the absence of CNF), condensed tannins from black wattle (12 g) were added to a glass container, then furfuryl alcohol (8 g), formaldehyde solution (2.5 g), and distilled water (2.4 mL) were introduced and homogenized for 1 min with a glass rod. Then, diethyl ether (blowing agent, 2 g) was added and homogenized for another 1 min, and lastly, *p*-toluene sulfonic acid solution (4.4 g) was added and briefly mixed. The mixture was kept undisturbed while the exothermic reaction led to expansion and foam growth. The foams were then placed in an oven at 80 °C where they were held until reaching constant mass. For the CNF-reinforced foams, we followed the same procedure, but the mass related to formaldehyde and water (4.9 g) was replaced by CNF suspensions, from 0.01 to 1.5% (w/v).

### Foam Characterization

#### Morphology

Foam density was measured by gravimetry (dry mass/volume). Foam microstructure was investigated by scanning electron microscopy (SEM) and X-ray microcomputed tomography (micro-CT). Field-emission SEM was carried out in a Zeiss Sigma (VP, Germany) using an acceleration voltage of 1.5 kV. The samples were coated with a 4-nm gold/palladium layer using a Leica EM ACE600 high-vacuum sputter coater. Micro-CT was utilized to assess the pore distribution, wall size, and overall morphology of the foams. The samples were scanned on a SKYSCAN 1272 device (Bruker micro-CT, Belgium) under a source voltage of 20 kV and a current of 175 μA. The three-dimensional (3D) projections were reconstructed using the NRecon 1.6.10.4 software, and the images were analyzed in the CTVox 3.3 (3D viewing and slicing), CTAn 1.13 (morphometric parameters), and DataViewer 1.5.6.2 (2D viewing) softwares, all from Bruker micro-CT. At least three samples of each treatment were analyzed, and within a sample, at least three volumes of interest were selected for quantitative treatment. For the pore size and wall thickness, at least 200 measurements were averaged.

#### Mechanical Properties

The mechanical strength of the foams was evaluated by uniaxial compression using a TA.XTplusC Texture Analysis. The measurements were taken at a compression rate of 0.10 mm s^–1^, based on preliminary tests, and a conditioned environment at 23 °C and 50% relative humidity (RH).

#### Thermal Insulation

We used an infrared camera (FLIR, T620Bx model) to assess the thermal insulation of the foams. In a typical procedure, the samples were placed on a heating source set to 100 °C, and the evolution of the thermal profile was recorded for 2 min with the thermal camera. Prior to the test, the samples were conditioned under different RH conditions using pure water and saturated MgCl_2_ and NaBr to achieve 33 and 58% equilibrium RH, respectively. Oven-dried samples (100 °C until constant mass) were also utilized. Sample temperature was extracted from the infrared video captured at 20 fps by determining the temperature values every 10 frames within a manually defined region extending the full height of the sample using Matlab R2019b (MathWorks). A five-point moving average was then applied to the mean pixel value graphs at constant height relative to the heat plate surface.

#### Fire Retardancy

The fire retardancy and self-extinguishing properties of the foams were assessed using a homemade flame test and by thermogravimetry (TG). The samples were positioned in a metallic holder placed on a scale and exposed to a flame (from butane, characteristic temperature of over 1500 °C) for 120 s. The flame and sample were separated by 5 cm from each other. The mass loss in all samples was recorded during 120 s and used to determine the kinetic profile and the total mass loss. TG was carried out on a Q500 equipment (TA Instruments) under air atmosphere (10 mL min^–1^) from 50 to 800 °C at a 10 °C min^–1^ heating rate.

## Results and Discussion

### Morphology and Mechanical Performance

A typical tannin-based rigid foam is formed from the polymerization of condensed tannins with furfuryl alcohol (FA) using acid as catalyst. Chemical crosslinkers are typically used to strengthen and toughen the cell wall of the foams, given that the resulting tannin-FA copolymer is typically brittle. Several crosslinkers have been tested over the years,^18^ with formaldehyde consistently showing one of the best performances. Therefore, we used formaldehyde as a benchmark crosslinker in our reference sample. In the precursor mixture, we replaced the crosslinker by CNF at given mass concentrations, ranging from 0.1 to 1.5 wt %. Such addition is equivalent to a nanofiber fraction ranging from 0.002 to 0.36% based on the dry mass of the final foam. To favor the exothermic polymerization reaction, we acclimatized the CNF suspensions at 80 °C for 30 min prior to the foam formation. This warranted a homogenous foam growth, which was not observed when using cold (∼4 °C) CNF suspensions (data not shown).

**Figure 2:**
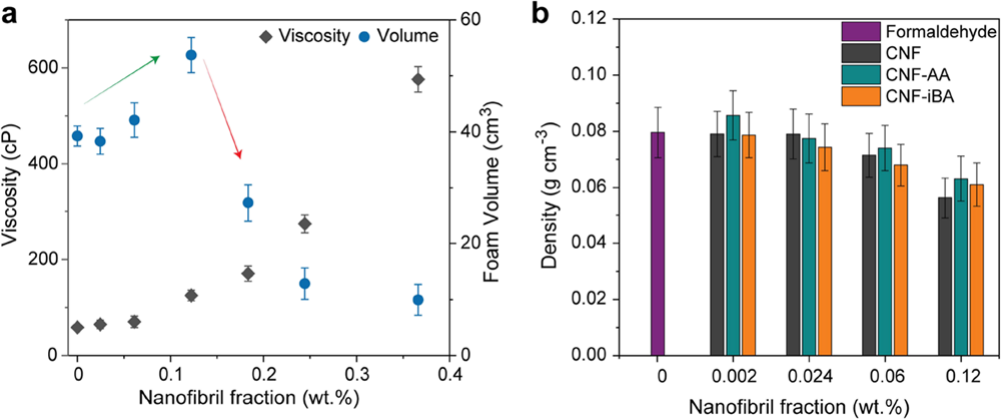
(a) Effect of the cellulose nanofibers (CNF) added at different concentrations on the viscosity of the foam precursor and as-produced foam volume. (b) Density of the foams calculated as a function of the nanofiber fraction and the CNF type, compared to the reference (formaldehyde-crosslinked) foam.

In tannin-FA rigid foams, the pores and overall foam structure are formed from bubbling a blowing agent that is added to the precursor. Here, we use diethyl ether, but we point out that several other greener molecules have been used efficiently as well.^14,19^ As far as foam growth, one should consider the viscosity of the precursor medium, that is, solvent bubbling and its removal by evaporation cannot lead to pore formation at high viscosity. CNF suspensions are known to form highly viscous gels at low mass fractions (∼1.5 wt %) because of the entanglement and interconnectivity of the nanofiber network.^27^ The addition of CNF at low loading increased the volumetric foam growth (Figure 2a), which is due to the dispersive character of CNF.^28^ However, when the nanofiber fraction reaches *ca.* 0.2%, the viscosity of the system is too high (*ca.* 300 cP), leading to a reduced foam growth and to dense CNF-tannin-FA foams (Figure 2b). Therefore, we used 0.12% nanofiber as the maximum fraction (correspondent to the addition of a CNF suspension at 0.5 wt % in the precursor), which led to a foam density of *ca.* 0.06 g cm^–3^ (Figure 2b). The foams obtained from unmodified CNF and esterified counterparts (less hydrophilic CNF-AA and CNF-iBA) presented similar density.^23^ The nanocellulose loading is probably too low to lead to significant changes in density. When compared to the reference foam, the CNF-loaded foams were less dense, highlighting the dispersion capability of CNF, also demonstrated in stabilizing O/W multiphase systems.^29,30^

**Figure 3:**
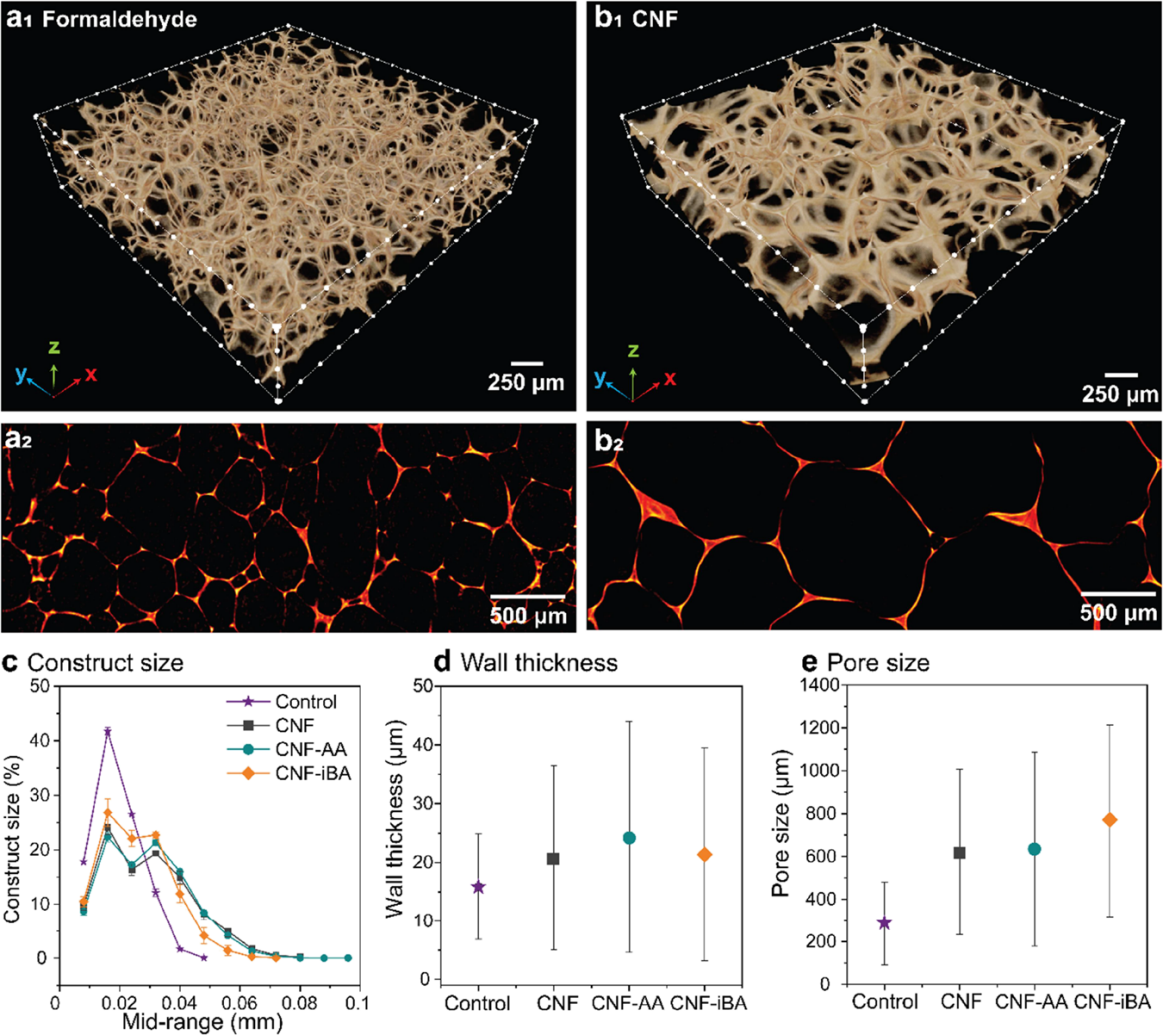
Microstructure of the CNF-reinforced foam compared to the reference foam (prepared by crosslinking with formaldehyde). 3D reconstructed images (a_1_ and b_1_) and 2D slices (a_2_ and b_2_) of the foams prepared with formaldehyde crosslinker (a) and CNF reinforcement (b). Image analysis by using the measured volume in a given size range (c), average values of pore wall thickness (d), and pore size (e). Distributions and all measured features are shown in Figures S3–S5.

**Figure 4:**
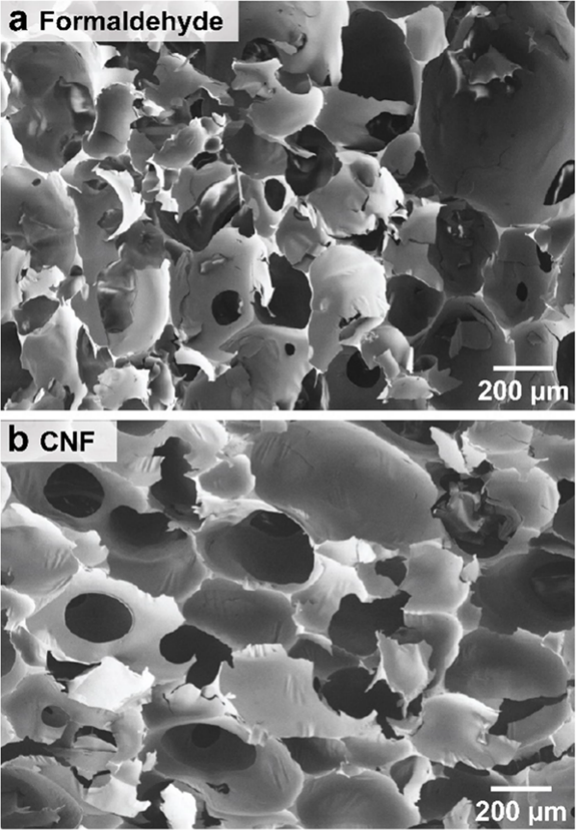
SEM images of the (a) reference foam (crosslinked with formaldehyde) and (b) CNF-reinforced tannin-based rigid foam.

The addition of CNF to the tannin-FA formulation did not change the chemical structure of the resulting tannin-FA polymer (Figure S2). However, CNF remarkably changed the morphological features of the resulting foams (Figures 3, 4, and S4). Foams with the pore size in the submillimeter length scale were obtained (Figure 3e), having most of the construct size (including cell corners) in the 10–30 μm range, as shown by the peak at the mid-range of 0.02 mm (Figure 3c) and the average values for wall thickness (measured at the cell–cell interconnection) around a similar range, 15–25 μm (Figure 3d). Foams prepared with the chemical crosslinker displayed smaller structural elements, both pore size (averaging 300 μm) (Figure 3e) and wall thickness (15 μm), compared to the foams in the presence of CNF. It should be noted that the size distribution in both cases is rather wide. The average wall thickness and the pore size of the foams carrying CNF were 33–66 and >130%, respectively, higher than those observed for the reference foam. The increased size was affected by the CNF type and was not proportional as far as wall/pore relationship, thus explaining the lower density of the foams that contained CNF compared to the reference. Finally, decreasing the hydrophilicity of the CNF led to a slight increase in the pore size of the foams, whereas the chemically crosslinked foams (via formaldehyde) showed a more homogenous pore distribution and smaller characteristic sizes.

**Figure 5:**
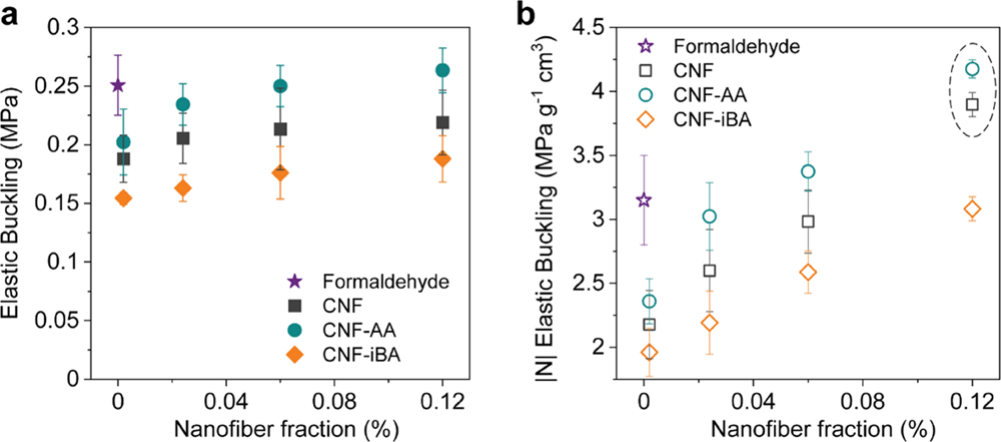
(a) Ultimate and (b) relative (normalized by density) strengths obtained from uniaxial compression assays of tannin-based rigid foams prepared with unmodified or esterified CNF (data are added for reference foams, for example, produced by formaldehyde crosslinking).

The resolution threshold of micro-CT is *ca.* 2–4 μm, at the given conditions, which may lead to erroneous conclusions as far as the pore windows thicknesses of the tannin-FA foams. Whereas the foams appear to have open cells in the micro-CT scans (Figure 3), the SEM images (Figure 4) reveal nearly fully reticulated (i.e., closed) cells. The central areas of the pore windows (the ones not showing in Figure 3) are expected to be below 2–4 μm (micro-CT resolution), whereas the pore corners are thicker and impart mechanical integrity to the foam. A lower mechanical performance track with large pores, but this is not the case of nearly or fully reticulated rigid foams, where the influence of the pore size on mechanical performance is less evident.^31^ In fact, closed cell rigid foams, with pores in the range measured with our materials, should lead to similar mechanical properties when the foam is 90–100% reticulated.^31^ Therefore, the gains in mechanical performance observed in our tannin-FA foams (Figure 5) are ascribed to the effect of CNF, for example, from cell wall reinforcement enabled by the fibrillar network with an interconnected structure. While covalent bonds are formed between the chemical crosslinker and tannin-FA polymer, nanocellulose interacts with the polyphenolics present in the cell wall of the foam by multiple and dynamic secondary interactions. Bond breaking/reforming lead to toughening, as typically observed in cellulose-based supramolecular constructs.^32^ Nevertheless, plant-based polyphenolics, such as tannins, interact with virtually any surface,^33−36^ thus promoting high adhesion at nanocellulose-tannin interfaces, which lead to an efficient stress-transfer mechanism from the polymer matrix onto the reinforcing nanofiber skeleton.

As seen in Figure 5, the addition of only 0.12% CNF led foams that are stronger (specific strength) compared to those crosslinked with formaldehyde (reference). All foams behave similarly in the elastic–plastic regime, having an abrupt breakage after the elastic zone at 2–3% strain. Overall, at the lowest nanofiber content (0.002%), all CNF-reinforced foams displayed lower elastic buckling compared to the reference foams; however, there was a gain in mechanical performance when CNF was added to the foam precursor. CNF-AA, that is, acetylated CNF at C6-OH, led to the materials with the highest mechanical performance, followed by native CNF. CNF-AA offered a more favorable interfacial interaction between the reinforcing nanofibers and the amphiphilic tannin-FA copolymer. Acetylation of plant fibers has proven to be efficient to enable efficient compositing with polyolefins.^27^ CNF-iBA, which is more hydrophobic than CNF-AA, led to the weakest materials. Therefore, the hydrophilicity–hydrophobicity balance of the reinforcing nanofibers should match the tannin-FA to optimize mechanical performance. Moreover, the addition of CNF to the foams led to lighter materials, resulting in materials with higher specific strength (nearly 35% increase) of what was observed for the chemically crosslinked foams (Figure 5b). The CNF-reinforced tannin-FA foams met the structural criteria for applications in roof insulation, which are the most demanding ones concerning structural integrity (Types I to III determined by D7425/D7425M normative).

### Thermal and Fire-Retardancy Performance

So far, we have shown that the addition of CNF to tannin-FA foam decreases density and strengthens the cell walls. These features led to CNF-reinforced foams that were both lighter and stronger than the chemically crosslinked (formaldehyde) analogues. The thermal insulation and fire resistance of such materials are key features for building applications. Heat insulating capacity is not expected to change for our CNF-reinforced tannin-FA foams, when compared to the reference, because their pore size range (hundreds of μm) falls within a window where most of the insulating capacity comes from the air pockets. This is confirmed by the thermal conductivity of tannin-FA foams, *ca.* 30–40 mW m^–1^ K^–1^,^18,37,38^ that is near the one measured for air, *ca.* 25 mW m^–1^ K^–1^. Moreover, the chemical composition of both foam types does not differ significantly, given the low CNF loading. Nevertheless, considering the hydrophilic character of CNF, we investigated the effect of humidity in CNF-reinforced foams as a function of the nanofiber surface chemistry.

**Figure 6:**
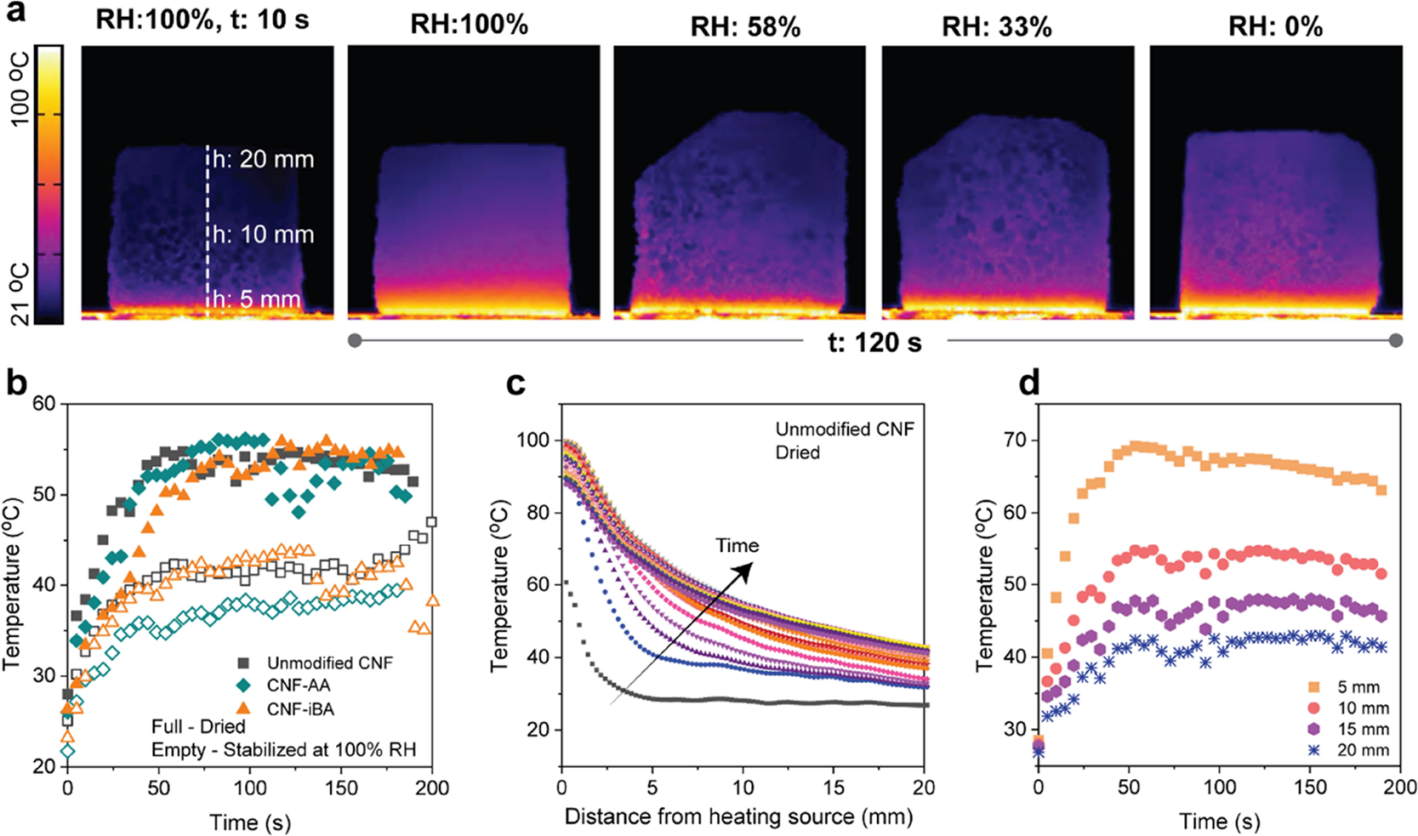
Assessment of the thermal insulation capacity of the CNF-reinforced tannin-based rigid foams and the effect of moisture content. (a) Thermal images of CNF-reinforced foams, equilibrated at a given relative humidity (RH), at 10 and 120 s after contact with a heating source at 100 °C. (b) Extract data for the temperature shift kinetics of the foams prepared with unmodified CNF and their esterified counterparts (CNF-AA and CNF-iBA) when dried or stabilized at 100% RH. (c) Temperature profile as a function of the distance from the heating source for the foams prepared with unmodified CNF in dry conditions. (d) Temperature profiles as a function of time for four given distances from the heating source (5, 10, 15, and 20 mm) for the foams prepared with unmodified CNF in dry conditions.

CNF-reinforced tannin-FA samples were placed on a heating source at 100 °C, and the thermal gradient across the foam was measured as a function of time with an infrared thermal camera (Figures 6 and S6–S8). The CNF type led to foams with a similar behavior in thermal insulation capacity, owing to the fact that the nanofibers are a minor component and the fact that CNF is embedded in the tannin-FA matrix. Therefore, the remarkably different interactions with water of esterified CNF, including water vapor, is shown to be only relevant for structuring purposes. The humidity content, however, had a major impact on the temperature gradient across the foam sample. Humidity in the sample led to cooling, as can be seen in Figure 6b, where moist samples stabilized the temperature at least 15 °C below what was observed for dried samples. Overall, the thermal stabilization process takes place very fast, at the first 20 s (Figure 6c), and only 20 mm of material is enough to stabilize the temperature at *ca.* 40 °C (Figure 6d). The CNF-reinforced tannin-FA foams underwent 30 °C reduction of temperature with only a 5-mm-thick foam (Figure 6d), which can lead to very efficient insulation materials.

**Figure 7:**
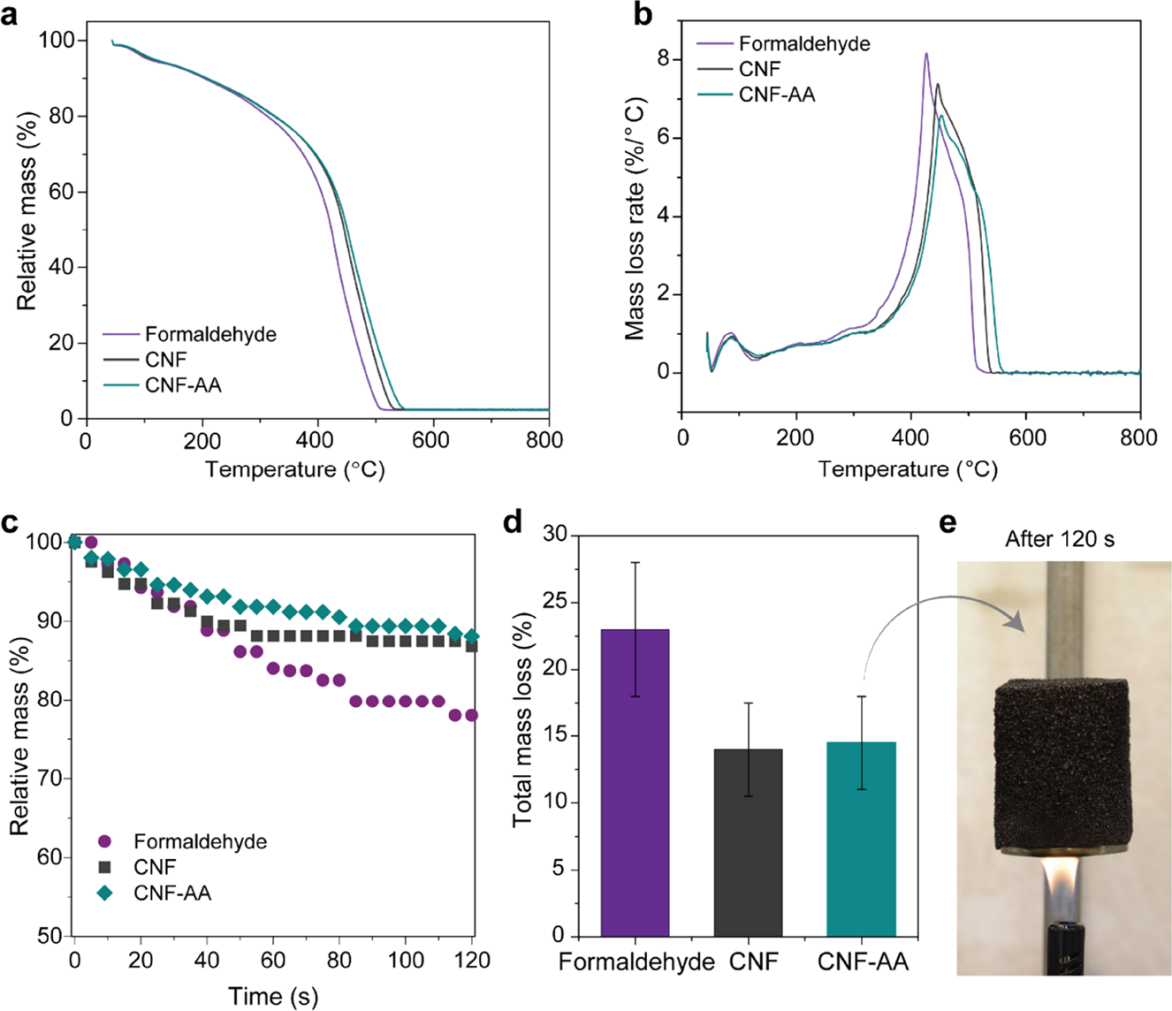
(a) TG and (b) derivative TG profiles (in the air atmosphere), indicating the mass loss of tannin-FA foams crosslinked with formaldehyde or reinforced with CNF (unmodified or esterified). (c) Mass loss profile of foams exposed to direct butane flame and (d) total mass loss after 120 s. (e) Visual appearance of the foam under direct contact with the flame; no ignition is seen after 2 min.

Lastly, we evaluated the mass losses of foams exposed to heating under an oxidative atmosphere (Figure 7a, b) and upon direct contact with a butane flame (Figure 7c–e). CNF foams showed better thermal resistance compared to the chemically crosslinked reference, as indicated by the mass loss peak with a *ca.* 20 °C shift. This is a result of formaldehyde oxidative reactions, indicating that the crosslinker is neither completely reacted nor removed from the sample during the thermal treatment of the foams after curing. Additionally, formaldehyde is volatile at room temperature. By contrast, CNF is thermally stable up to 300 °C.^39^ This endows the CNF-reinforced foams with a higher decomposition temperature and helps to stabilize the system during the formation of highly thermally stable char layer, which is known to improve fire resistance. We observed the same behavior when exposing the foams to a butane flame (Figure 7c–e). The formaldehyde foams burned faster, produced a more intense flame, and underwent mass losses of *ca.* 22%. The CNF-reinforced foams showed a mass loss of *ca*. 12% after *ca*. 80 s exposure to the flame (Figure 7c). Formaldehyde has been banned from several applications, given its hazardous effects to mammals.^40^ Meanwhile, cellulose is inert and safe, and its pyrolysis does not generate any toxic compounds.^27^ CNF-reinforced tannin-FA foams are self-extinguishing, showing no ignition along several minutes of exposure to a direct flame (Figure 7e).

### Benchmarking and Feasibility of CNF-Reinforced Tannin Foams

Technical feasibility and economic viability are factors to consider whenever an investigation is devoted to replacing a well-established component in a formulation, for instance, by less-expensive or greener analogues. Compared to formaldehyde, the model crosslinker taken as benchmark, CNF is both greener and more cost effective in relative terms. Even if the price per dry ton of CNF (USD 1800–2400)^41^ is higher than that of formaldehyde (the 37% solution typically ranges in price from USD 400–800), the contents required in the formulations differ in more than one order or magnitude: 1.2 kg CNF per ton of foam (dry basis), as shown herein, ensures technical feasibility in terms of performance; *ca.* 80 kg of 37% formaldehyde solution is the typical amount added to produce 1 t of foam. Accounting for market fluctuations and overestimating CNF cost to USD 2500 t^–1^ while underestimating formaldehyde cost to the lowest level would lead to additive costs per ton of foam of *ca.* USD 3 in the case of CNF and USD 30 for formaldehyde, again in one order of magnitude difference.

Tannin foams are herein compared in terms of performance to benchmark the most relevant properties, for instance, as far as thermal insulators. Although typically delivering higher thermal conductivities than the commercially widespread polystyrene and PF, tannin foams—including our CNF/tannin foams—are flame self-extinguishing while the references above are flammable and known to release toxic gases. Although all being highly porous, the density of tannin foams (0.06–0.08 g cm^–3^), either containing CNF or not, are typically not as low as those of commercial polystyrene (0.015–0.05 g cm^–3^) and PF (0.02–0.06 g cm^–3^). However, the compression strength of CNF-reinforced tannin-based foams (0.15–0.30 MPa) is remarkably higher than the typical range of expanded polystyrene foams (0.04–0.12 MPa) and comparable to extruded polystyrene (0.1–0.7 MPa) and polyurethane (0.2–0.4 MPa) foams.^37,42^

## Conclusions

Isocyanate-free foams are produced from tannins extracted from the bark of black wattle tree, which are widely available renewable molecules derived from forest biomass. Rigid foams based on tannin are further reinforced with CNF, eliminating the need for chemical crosslinkers such as formaldehyde. CNF not only increases the biobased content of the foams but also reduces the density while enhancing the compressive strength. Remarkably, the CNF-reinforced foams show better performance in heat and flame resistance. Surface modification of CNF reduces its wettability and tailors the physical–mechanical properties of the reinforced foam by strengthening the cell walls of the foam. We show biobased solutions that produce gains in safety and performance, fire protection, and thermal insulation for passive heat/cold storage.

## Supporting Information Available

FTIR and water contact angle of CNFs; FTIR of the foams; additional 2D slices and 3D reconstructions of microtomography; data of temperature profile over time and distance; and properties of the tannin used (PDF)
