# Supplementary material for: Nanocellulose Removes the Need for Chemical Crosslinking in Tannin-Based Rigid Foams and Enhances Their Strength and Fire Retardancy
Source: ACS Sustain Chem Eng. 2022 Jul 25;10(31):10303–10. doi: 10.1021/acssuschemeng.2c02678 (PMC9364407; doi:10.1021/acssuschemeng.2c02678)
Supplement: Supplementary file 1 — sc2c02678_si_001.pdf [file sc2c02678_si_001.pdf]

SUPPORTING INFORMATION

Nanocellulose removes the need for chemical crosslinking in tannin-based rigid foams and enhances their strength and fire retardancy

André Luiz Missio[†§], *Caio G. Otoni*[‡§], *Bin Zhao*[ϱ], *Marco Beaumont*[⁑], *Alexey Khakalo*[£], *Tero Kämäräinen*[ϱ], *Silvia H. F. Silva*[†], *Bruno D. Mattos*[§,ϱ]*, *Orlando J. Rojas*[ϱ,⁑,*]

[†] Graduate Program in Materials Science and Engineering (PPGCEM), Federal University of Pelotas (UFPel), Gomes Carneiro 1, Pelotas, RS 96010-610, Brazil

[‡] Department of Materials Engineering (DEMa), Federal University of São Carlos (UFSCar), Rod. Washington Luís km 235, São Carlos, SP 13565-905, Brazil

[ϱ] Department of Bioproducts and Biosystems, School of Chemical Engineering, Aalto University, Vuorimiehentie 1, Espoo FI-00076, Finland

[⁑] Department of Chemistry, Institute of Chemistry of Renewable Resources, University of Natural Resources and Life Sciences, Konrad-Lorenz-Str. 24, 3430 Tulln, Austria

[£] VTT Technical Research Centre of Finland, P.O. Box 1000, Espoo FI-02044 VTT, Finland

[⁑] Bioproducts Institute, Department of Chemical and Biological Engineering, Department of Chemistry and Department of Wood Science, University of British Columbia, Vancouver, British Columbia V6T 1Z4, Canada.

This file contains 10 pages, 8 figures and 1 table.

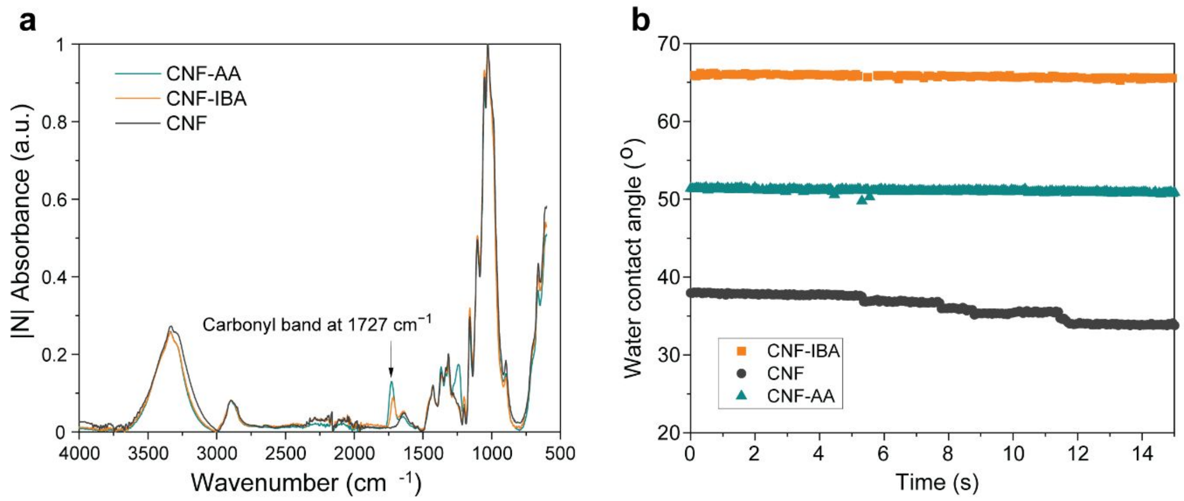

Figure S1. Fourier-transform infrared (FTIR) spectra and water contact angle profile of native (CNF) and esterified (CNF-AA and CNF-iBA) cellulose nanofibers (CNF).

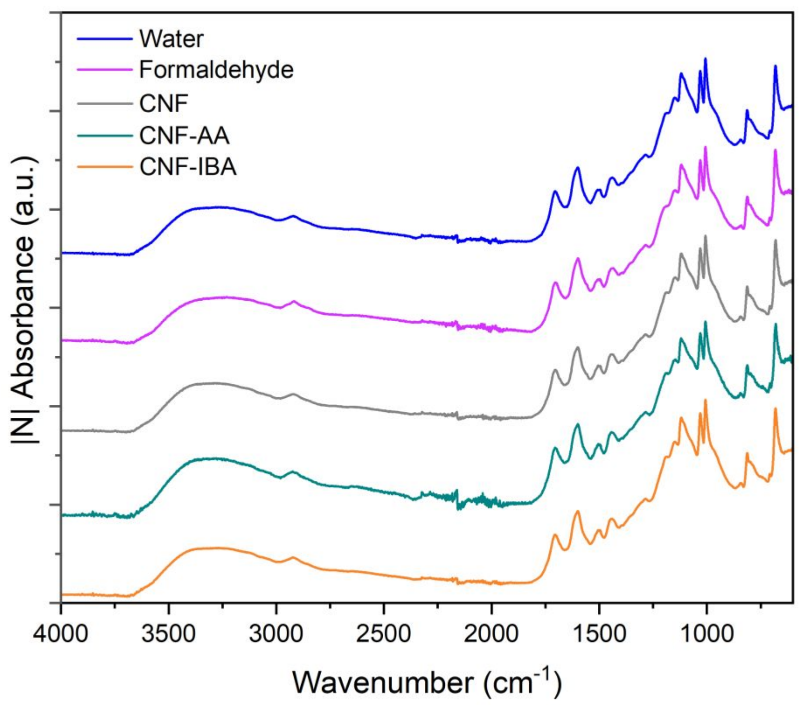

Figure S2. Fourier-transform infrared (FTIR) spectra of the tannin-based rigid foams: Control, chemical crosslinker-free samples are foams prepared with water replacing formaldehyde, while traditional foams prepared with formaldehyde as benchmark. CNF-reinforced foams contained 0.12 wt% of nanofiber fraction over the total mass of the foam.

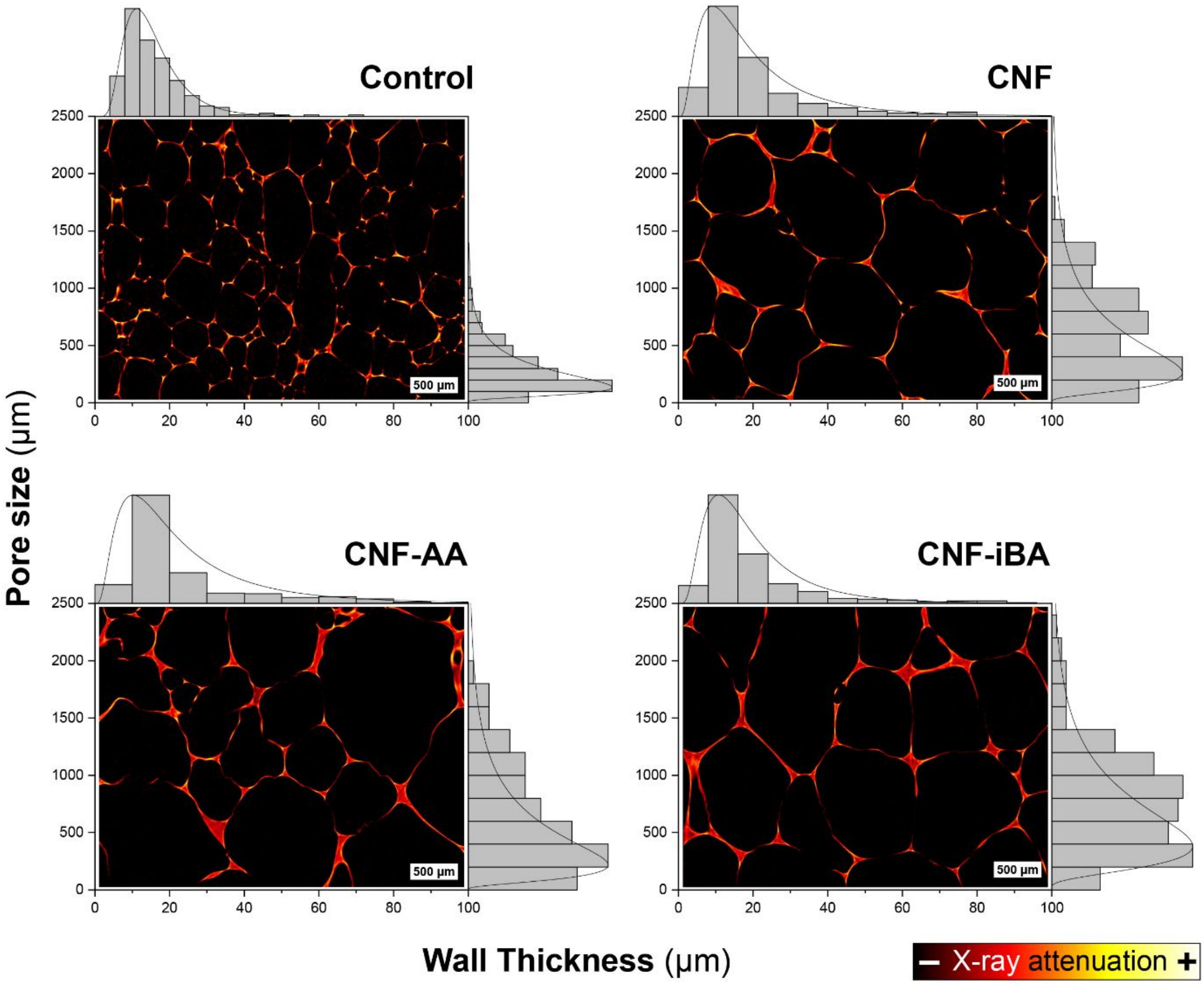

Figure S3. 2D reconstructions of formaldehyde-crosslinked (Control) and cellulose nanofiber (CNF)-reinforced tannin-based rigid foams relying on the differential attenuation of X-ray between voids and cell walls. The dimensions of the pore (Y-axis) and wall (X-axis) are plotted as count-based histograms (n ≥ 200). CNF was either pristine or regioselectively esterified with acetyl (C6AA-CNF) or isobyturyl (C6BA-CNF) moieties.

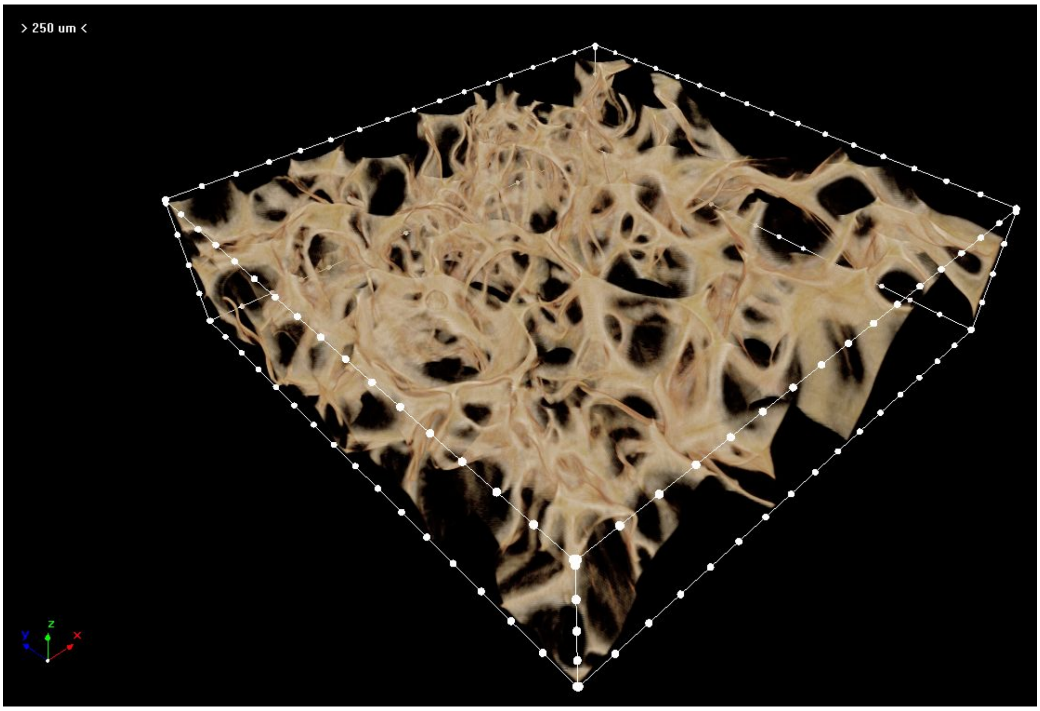

Figure S4. 3D micro-CT reconstructions of tannin-based rigid foams reinforced with acetylated cellulose nanofibers. The lighter, the greater the X-ray attenuation.

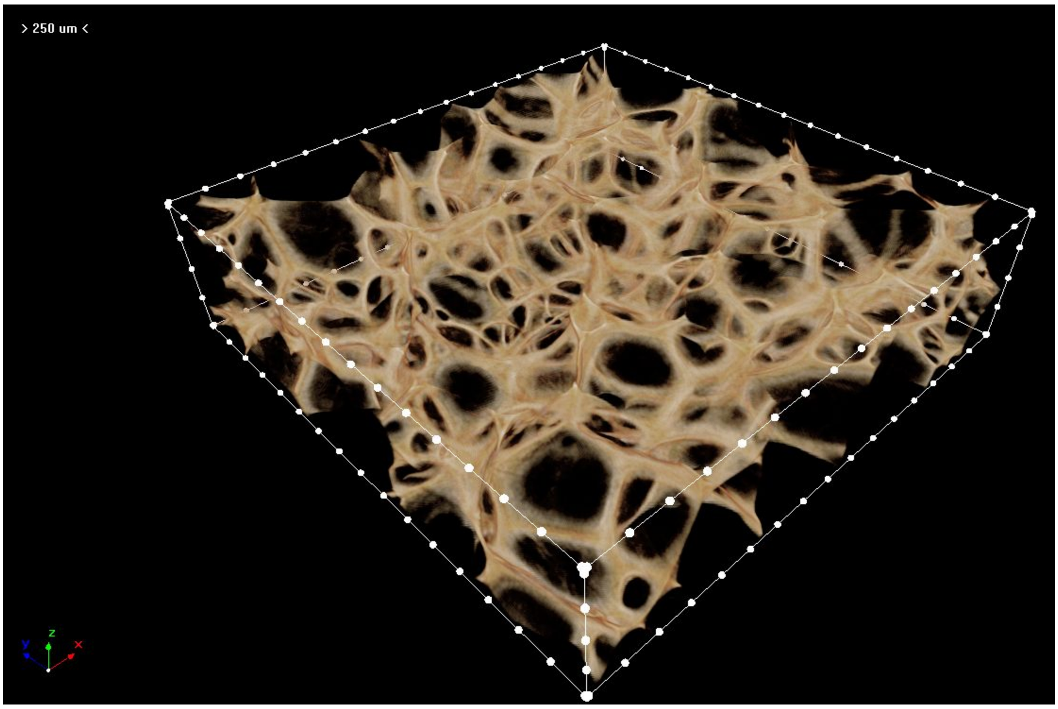

Figure S5. 3D micro-CT reconstructions of tannin-based rigid foams reinforced with isobutyrylated cellulose nanofibers. The lighter, the greater the X-ray attenuation.

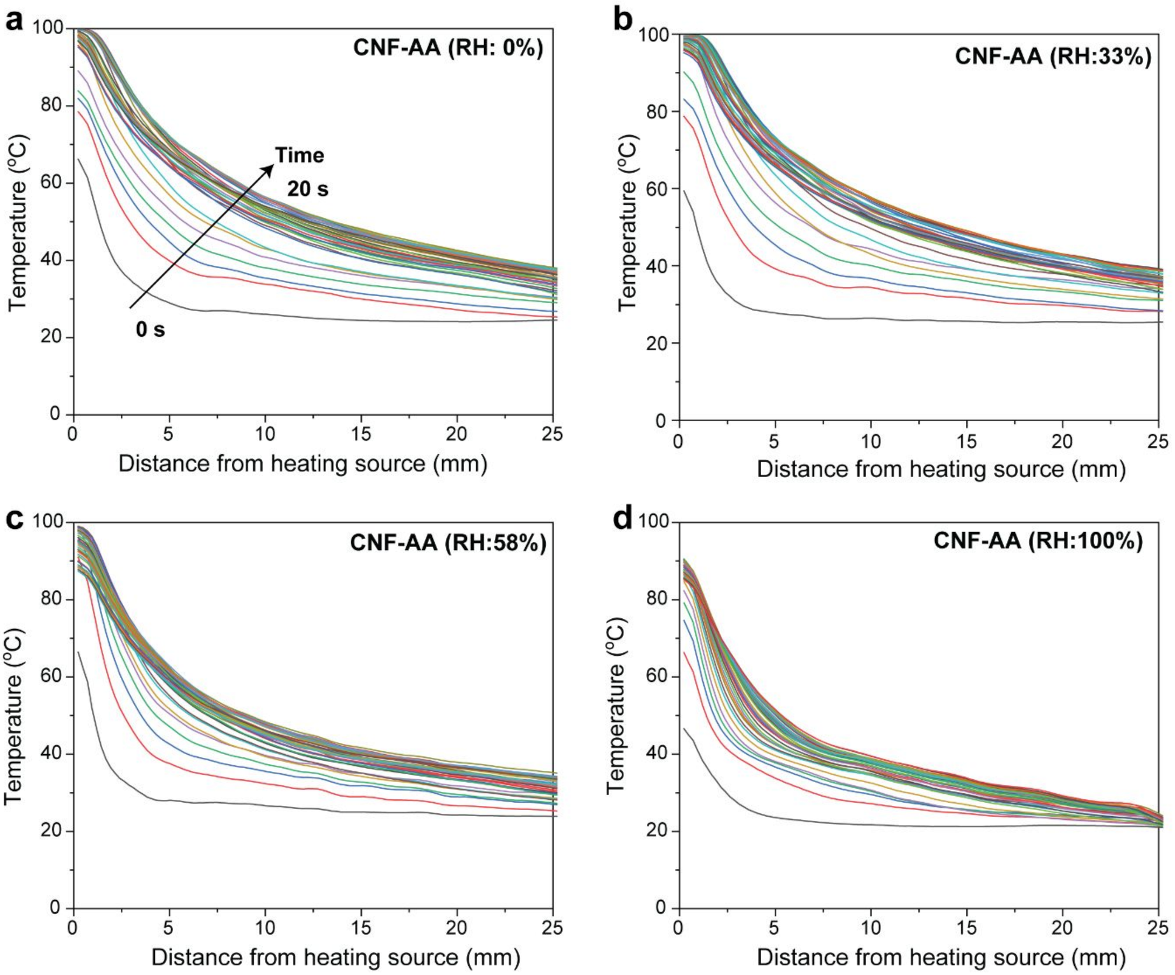

Figure S6. Temperature profiles as a function of the distance from the heating source for the foams prepared with acetylated cellulose nanofibers (CNF-AA) at varying relative humidity conditions.

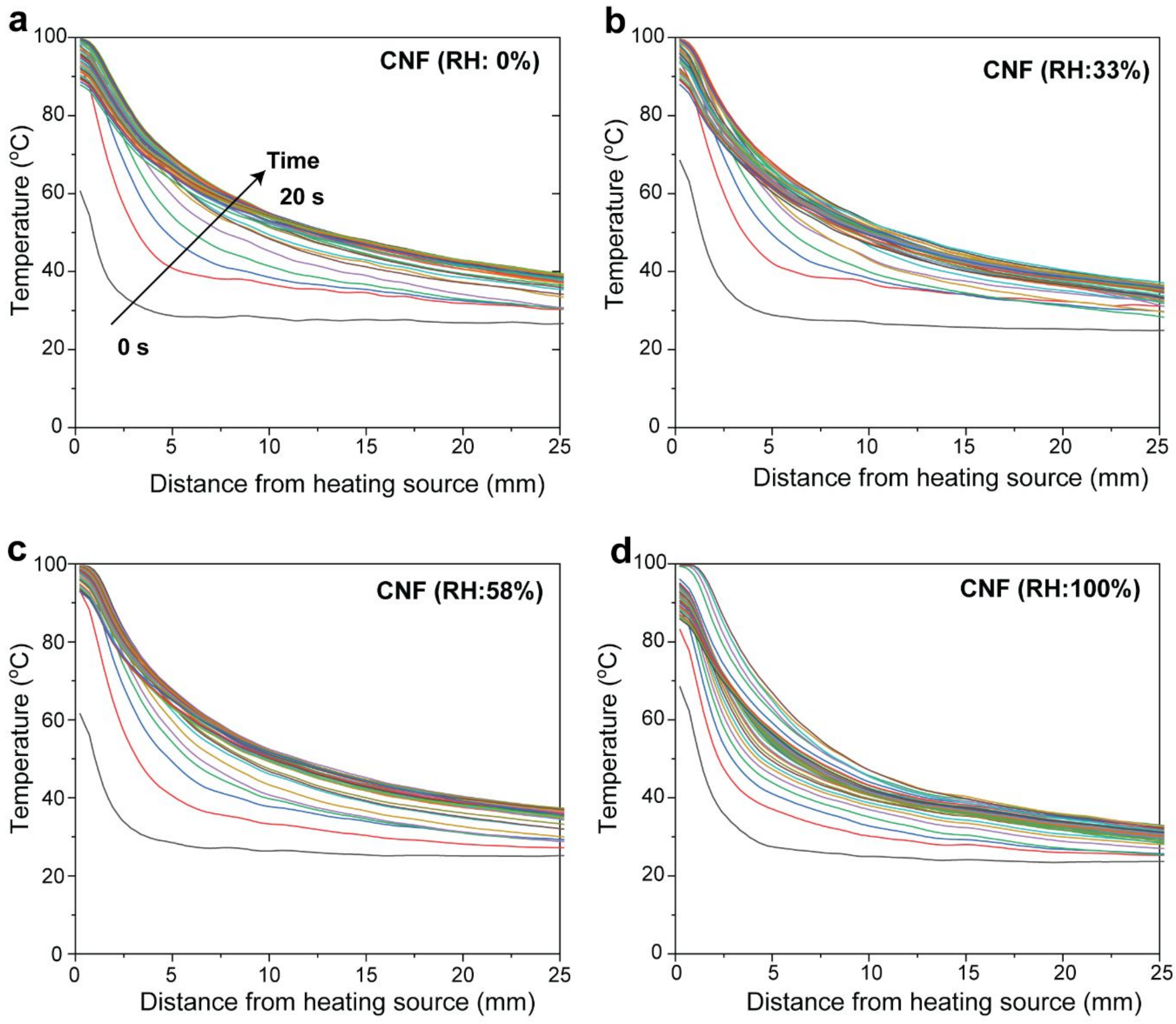

Figure S7. Temperature profiles as a function of the distance from the heating source for the foams prepared with pristine cellulose nanofibers (CNF) at varying relative humidity conditions.

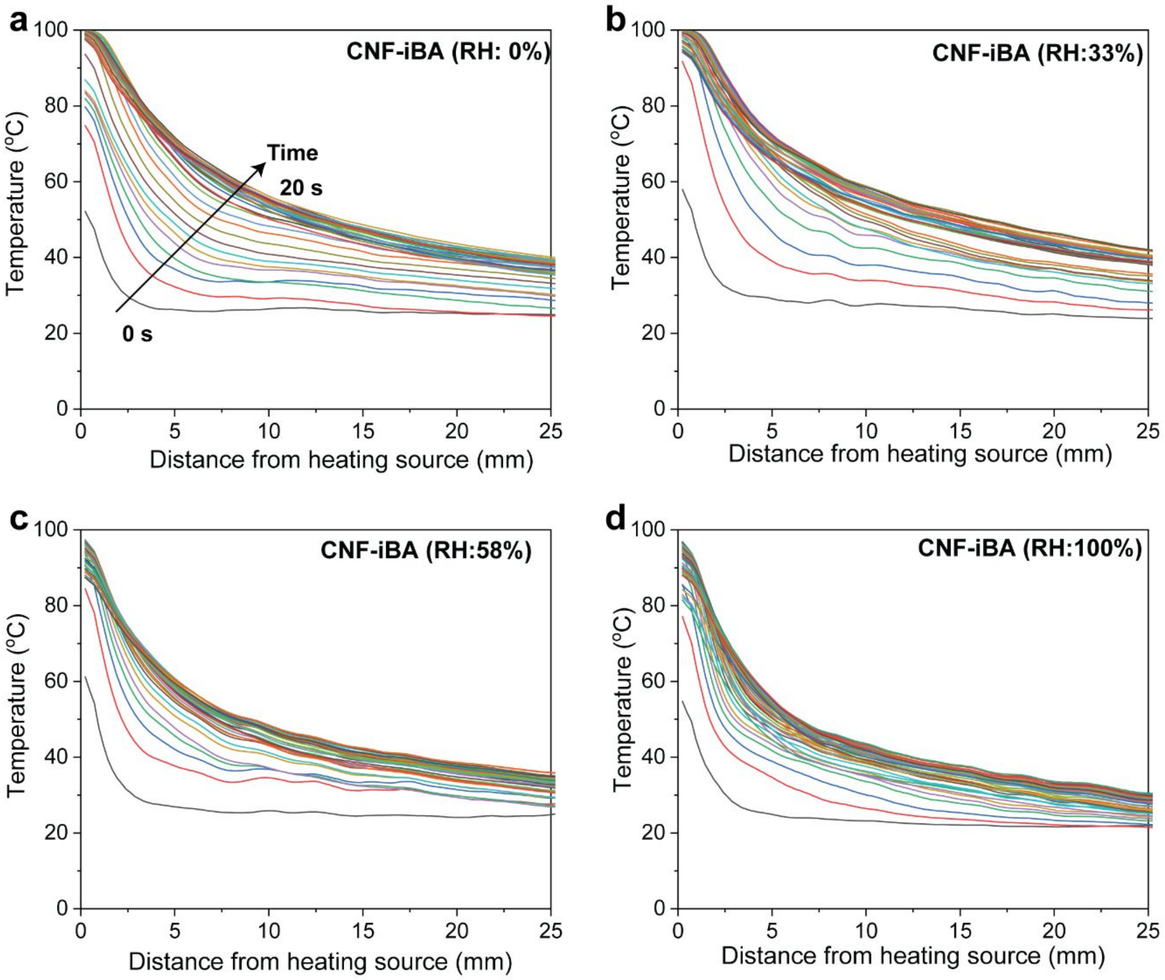

Figure S8. Temperature profiles as a function of the distance from the heating source for the foams prepared with isobutyrylated cellulose nanofibers (CNF-iBA) at varying relative humidity conditions.

Table S1. Properties of condensed tannins

Property	Value
Equilibrium moisture content (%)	9.9 ± 0.4
Content of ashes (%)	5.2 ± 0.2
Total phenolic content (mg GAE/g)	869 ± 6
Condensed tannins (mg CE/g)	410 ± 5
ORAC (μmol TE/g)	4934 ± 59
M_w (g/mol)	2639
M_n	670
